# Supplementary material for: Nitrite Enhances MC-LR-Induced Changes on Splenic Oxidation Resistance and Innate Immunity in Male Zebrafish
Source: Toxins (Basel). 2018 Dec 3;10(12):512. doi: 10.3390/toxins10120512 (PMC6315824; doi:10.3390/toxins10120512)
Supplement: Supplementary file 1 [file toxins-10-00512-s001.pdf]

Toxins **2018**, *10*, 512; doi:10.3390/toxins10120512

Supplementary Materials: Nitrite Enhances MC-LR-Induced Changes on Splenic Oxidation Resistance and Innate Immunity in Male Zebrafish

Wang Lin, Honghui Guo, Lingkai Wang, Dandan Zhang, Xueyang Wu, Li Li, Dapeng Li and Rong Tang

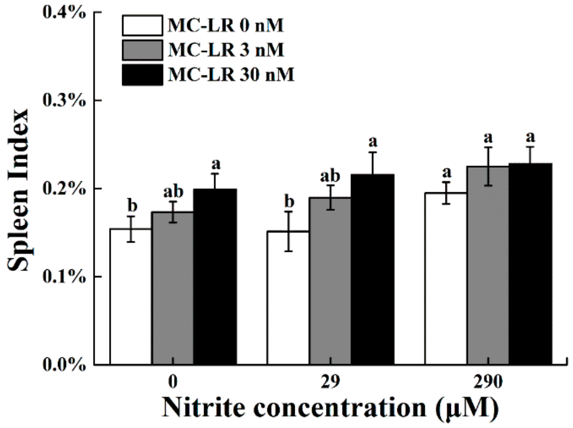

Figure S1. Alterations in the spleen index of male zebrafish exposed to different combinations of nitrite and microcystin-leucine arginine (MC-LR) for 30 d. Different letters above bars represent significant differences ($p < 0.05$).

Table S1. Measured concentrations of nitrite and MC-LR in water samples.

Nominal Concentrations		Measured Concentrations of Nitrite (μM) and MC-LR (nM) [a]
**Nitrite (μM)**	**MC-LR (nM)**	
0	0	< MDL [b], < MDL [c]
0	3	< MDL [b], 3.06 ± 0.26
0	30	< MDL [b], 30.88 ± 2.62
29	0	30.3 ± 3.04, < MDL [c]
29	3	29.4 ± 2.61, 3.12 ± 0.23
29	30	29.9 ± 2.32, 29.81 ± 2.98
290	0	303.3 ± 28.8, < MDL [c]
290	3	287.5 ± 29.4, 3.17 ± 0.22
290	30	296.1 ± 29.3, 30.89 ± 2.59

[a] Values are expressed as mean ± standard error (SEM). [b] MDL = minimum detection limit of nitrite (0.29 μM). [c] MDL = minimum detection limit of MC-LR (0.1 nM).

Table S2. Spearman correlation coefficients (r) between antioxidant parameters and innate immune parameters in male zebrafish after exposure [a].

Parameters	MDA	T-AOC	GSH	*cat1*	*sod1*	*gpx1a*
C3	-0.53**	0.62**	0.15	0.15	0.26	0.74**
il1β	-0.39**	0.55**	0.26	0.27	0.51**	0.53**
ifnγ	-0.50**	0.45**	0.17	0.25	0.42**	0.57**
tnfα	-0.51**	0.52**	-0.09	0.56**	0.41**	0.68**
c3b	-0.17	0.23	-0.05	0.57**	0.45**	0.46**
lyz	-0.09	0.19	-0.03	0.56**	0.59**	0.46**

[a] Analysis was conducted separately with 54 samples. $p < 0.01$ (**) indicate significant correlation between parameters. MDA, malondialdehyde; T-AOC, total antioxidant capacity; GSH, glutathione.

Table S3. Primer sequences used for real-time PCR.

Target Gene	Accession No.	Primer Sequences (From 5′ to 3′)	Product Length (bp)	Amplification Efficiency (%)
cat1	BC051626	F: CAAGGTCTGGTCCCATAAA<br>R: TGACTGGTAGTTGGAGGTAA	227	97.6%
sod1	BC055516	F: GTCCGCACTTCAACCCTCA<br>R: TCCTCATTGCCACCCTTCC	217	99.4%
gpx1a	BC083461	F: AGGCACAACAGTCAGGGATT<br>R: CAGGAACGCAAACAGAGGG	241	102.3%
c3b	AF047414	F: CAGTGGGAATATGTTGGCATTG<br>R: TTAGCTGCCCTTCATAACCTGTT	76	96.6%
lyz	NM_139180	F: AGGCTGGCAGTGGTGTTTTT<br>R: CACAGCGTCCCAGTGTCTTG	70	100.5%
il1β	AY340959	F: CATTTGCAGGCCGTCACA<br>R: GGACATGCTGAAGCGCACTT	63	98.5%
tnfα	AY427649	F: CCATGCAGTGATGCGCTTT<br>R: TTGAGCGGATTGCACTGAAA	68	104.4%
ifnγ	AY135716	F: GAATGGCTTGGCCGATACAGGATA<br>R: TCCTCCACCTTTGACTTGTCCATC	137	93.1%
gapdh	BC095386	F: CTGGTGACCCGTGCTGCTT<br>R: TTTGCCGCCTTCTGCCTTA	150	98.2%

Text S1. Pathological studies

Light Microscopic Observation

For the light microscopic study, spleens were first fixed in 10% neutral buffered formalin. After 24 h, samples were dehydrated in 70% ethanol for 30 min, 80% ethanol for 30 min, 95% ethanol for 30 min and 100% ethanol for 15 min. Then, samples were hyalinized in a mixture of xylene and ethanol (*v*/*v*, 1:1) for 15 min and 100% xylene for 15 min. After being immersed in paraffin wax for 60 min at 58 °C, spleen samples

were embedded, sectioned (5 μm) and stained with hematoxylin and eosin (H&E). Histopathological assessment was done on a Nikon H600L Microscope (Tokyo, Japan).

Transmission Electron Microscopic Observation

For the transmission electron microscopic study, samples were diced into 1 mm^3, prefixed in 2.5% glutaraldehyde solution, followed by three 15 min rinses with a 0.1 M phosphate buffer solution (PH 7.4). Post-fixation was in cold 1% aqueous osmium tetroxide for 1 h. After rinsing with the phosphate buffer again, the specimens were dehydrated in a graded ethanol series of 50–100% and then embedded in Epon 812 (Shell Chemical Co., NY, US). Ultra-thin sections were sliced with glass knives on an LKB-V ultramicrotome (Nova, Sweden), stained with uranyl acetate and lead citrate before examination under a HITACHI, HT-7700 electron microscope (Hitachi, Tokoyo, Japan).
